# Examining the impact of differing caffeine dosages in conjunction with plyometric training on physiological adaptations in basketball players

**DOI:** 10.1038/s41598-024-66275-8

**Published:** 2024-07-06

**Authors:** Siyuan Wu, Han Jiang

**Affiliations:** School of Physical Education, Wuhan Sports University, Wuhan, 430079 Hubei China

**Keywords:** Power training, Team sport, Aerobic power, Sports nutrition, Anaerobic power, Physiology, Metabolism

## Abstract

The aim of the current study was to investigate the effects of ingesting different dosages of caffeine (CAF) prior to plyometric jump training (PJT) on sport-related performance and physiological parameters in male basketball players. Twenty-four young athletes were randomly divided into 3 groups and performed 6 weeks of PJT while consuming 3 mg·kg^–1^ of body mass caffeine (CAF3, n = 8), 6 mg·kg^–1^ body mass caffeine (CAF6, n = 8) or placebo (PL; n = 8) one hour prior to each training session. Before and after the 6-week PJT, the players were evaluated for field-based basketball-specific performance measures (vertical jump, 20-m sprint, Illinois change of direction speed [CODS], and maximal strength) and lab-based physiological (aerobic capacity and anaerobic power) parameters. CAF3, CAF6, and PL groups demonstrated significant improvements in vertical jump (ES = 1.07, 1.45, and 1.1, respectively), 20-m sprint (ES = – 0.50, – 0.61, and – 0.36), change of direction performance (ES = – 1.22, – 1.26, and – 1.09), maximal strength (ES = 1.68, 2.29, and 1.17), maximum oxygen uptake (V̇O_2max_) (ES = 1.09, 1.59, and 0.92), and peak (ES = 1.82, 1.85, and 0.82) and average power output (ES = 1.39, 1.32, and 1.07) after 6 weeks of training. Comparative analysis of individual adaptive responses to training indicated that the CAF6 led to insignificantly greater effects in vertical jump (ES = 1.45), maximal strength (ES = 2.29), and V̇O_2max_ (ES = 1.59) with lower residuals in individual changes and lower coefficient of variations (CV) in mean group changes. Regarding sprint and CODS performance, both experimental groups indicated similar changes, residuals in individual changes, and CVs in mean group changes. Overall, consuming 6 mg·kg^–1^ body mass caffeine induces superior adaptations in aerobic fitness, anaerobic power, and sport-specific performance measures, with lower inter-individual variability in the adaptations and more homogenized changes over the training period.

## Introduction

Optimizing explosive power, sprint performance, and maintaining the ability to perform multiple sprints consecutively are considered important objectives of basketball-specific strength and conditioning programs^1^. This emphasis is based on the specific match movements players frequently engage in defensive and offensive situations, such as change of direction, sprints, and vertical and horizontal jumps^2^. In addition to these aspects, maintaining adequate balance and strength is crucial for basketball players to effectively execute multi-directional, high-intensity actions during the match^3,4^. Therefore, it is essential to develop training programs that effectively enhance these variables to augment players' physical ability on the court^1,4^.

There are various training approaches utilized by basketball players to enhance their explosive power, jumping ability, sprinting speed, and muscular strength^5–8^. Among these approaches, plyometric jump training (PJT) appears to be particularly prevalent and is one of the commonly used approaches by basketball players^7,9,10^. The utilization of the stretch–shortening cycle (SSC) in PJT involves the eccentric stretching of musculotendinous units during the loading or impact phase, followed by concentric shortening in the push-off or take-off phase^9^. This approach has proven to be an effective training method to enhance sport-related physical fitness, including sprinting speed, jumping ability, and change of direction, which are necessary for a successful basketball match^11,12^. Implementing PJT with basketball players boosts their physical performance and enhances their proficiency in basketball-specific tasks like defensive maneuvers, shooting, and rebounding^11,13–15^. Following the principle of training specificity, it is recommended that basketball players incorporate PJT programs into their regular training regimen.

Apart from training strategies to optimize athletes' performance adaptations, a considerable number of athletes and coaches have incorporated sports supplements into their training routines to enhance their physical capabilities^16–18^. Nutrition and supplementation have proven to be essential in optimizing performance, particularly in sports requiring intense exercises like basketball. Although the understanding of how sports supplements can impact athletes' performance is continuously growing, it is widely recognized that they play a crucial role in enhancing sports performance in both short and long-term durations^19^. Of the used supplements by athletes, caffeine stands as an effective supplement for neuromuscular, metabolic, and cardiovascular functions, leading to enhanced technical and physical performance. Caffeine is particularly renowned for its acute and chronic impact on athletic performance^20^. Caffeine is a potent supplement that can boost energy expenditure after physical activity^20^. It is a widely consumed bioactive substance in our diet and is known to stimulate the central nervous system^21,22^. Furthermore, it is believed to enhance thermogenesis and the oxidation of fat^21^. In addition, supplementations with caffeine could increase the muscle fibers' contractile properties, enhancing maximal intensity exercise performance^23^. These caffeine-induced effects result in enhanced jumping ability^20^, sprint performance^24,25^, reactive agility and decision time^26^, anaerobic power^21^, strength and muscular power ^23^, sprint performance^24,25^, and endurance time to exhaustion^27^.

Apart from the acute effects of caffeine ingestion prior to physical exercise^23^, it seems that long-term caffeine ingestion before exercise training could induce more adaptive responses induced by training. However, the effects of long-term caffeine ingestion prior to PJT on athletic performance adaptations of basketball players are unknown.

Previous research has investigated the acute and chronic impact of caffeine on the physical performance of athletes using varied doses of caffeine per kilogram of body mass, leading to conflicting findings^20,21,23^. For instance, a dose of 3 mg·kg^–1^ of body mass (BM) has been found to be effective in enhancing strength and power^23^ and volleyball-specific performance and skills^20^, whereas male athletes experienced significant performance-related effects with a dose of 6 mg·kg^–1^ of BM^23,28^. A recent study by Nemati et al.^20^ demonstrated that consuming 6 mg·kg^–1^ of caffeine results in superior performance outcomes in volleyball-specific tests compared to the 3 mg·kg^–1^ of BM dose. Regarding the effects of caffeine ingestion, previous studies have typically utilized acute effects of a dose of 3 or 6 mg·kg^–1^ of BM^19–21,23^. However, the effects of 6 mg·kg^–1^ caffeine ingestion on basketball-related performance and physiological adaptations over a training period remain unclear.

Considering the positive performance-related effects of caffeine, such as increased motor unit recruitment, improved Na^+^–K^+^ pump response, and enhanced calcium release from the sarcoplasmic reticulum^21,23^, it may be appropriate to consider it as a suitable sports supplement for basketball players during the preparation phase of their training schedules. It is worth noting that no previous studies have examined the effects of caffeine ingestion prior to PJT on athletic performance adaptations, specifically in basketball players. Therefore, further study on this topic could be necessary to understand the potential benefits of caffeine in this context.

In addition to the importance of caffeine in enhancing athletic performance, it is crucial to emphasize the individual variability in responses to supplementation during the study^21^. Previous studies incorporating caffeine supplementation to assess exercise performance often focused on group outcomes^19–21,23^, neglecting the individual differences in how subjects respond to training and supplementation. Consequently, it is essential for a more comprehensive understanding of the potential benefits and individualized responses with the integration of caffeine intake before PJT in basketball-related performance.

Therefore, the main aim of this study was to assess the impact of consuming two different doses (i.e. 3 and 6 mg·kg^–1^) of caffeine for 6 weeks before performing a PJT on the basketball-related performance measures (vertical jump, 20-m linear sprint, Illinois change of direction speed, and lower body maximal strength) and physiological parameters (cardiorespiratory and anaerobic performance). Additionally, this investigation sought to examine the individual responses to the training and caffeine ingestion to identify the most effective adaptation approach by analyzing the inter-individual variability in adaptive responses.

## Methods

### Participants

**Figure 1:**
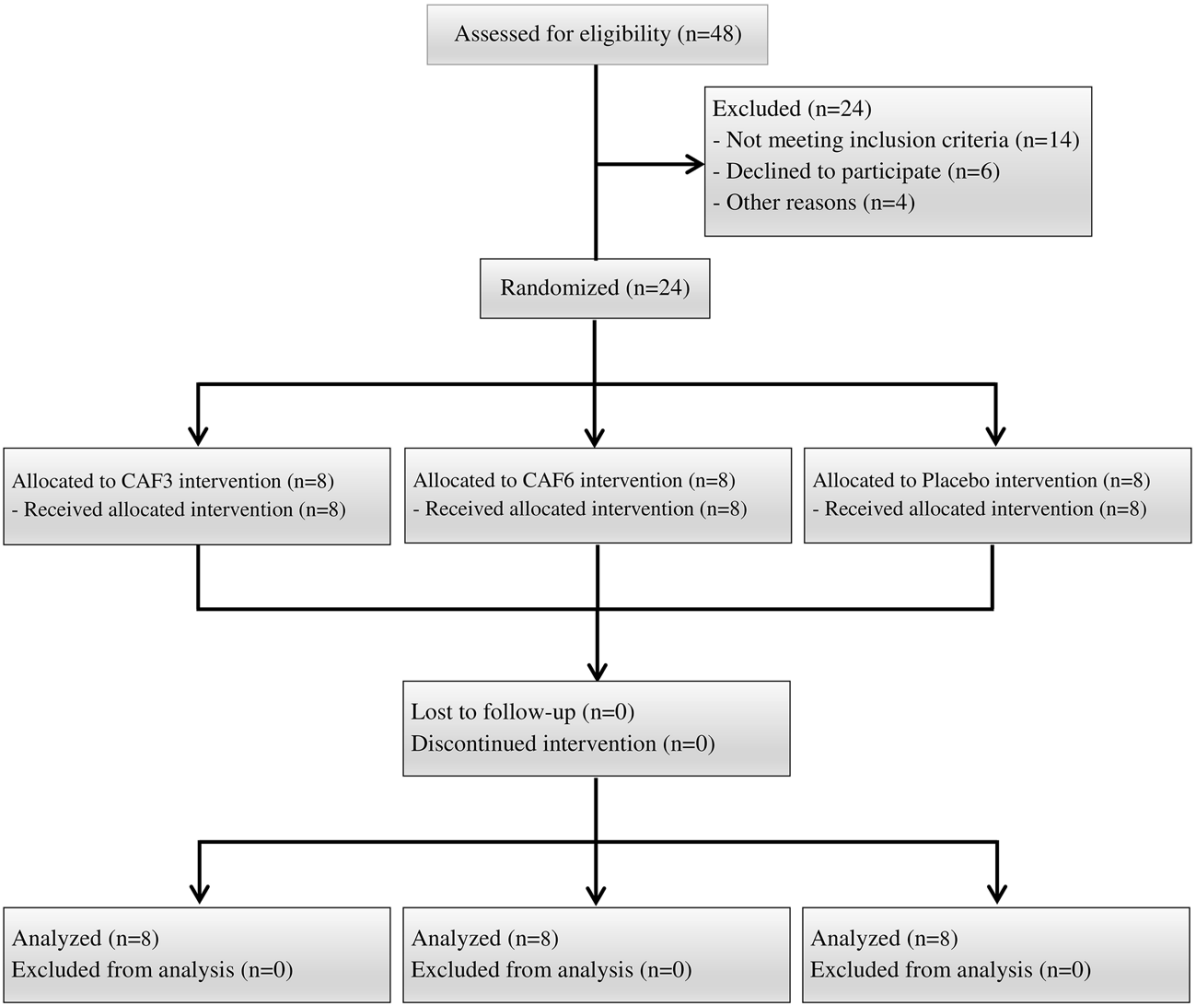
CONSORT flow diagram.

Using G*power software (Version 3.1.9.2, University of Kiel, Germany) with an alpha level of 0.05 and a power (1-beta) of 0.80, 8 participants were determined for each group to assess the effects of caffeine supplementation on the physiological parameters of basketball athletes^29^. Twenty-four male collegiate basketball players participated in the current study. Participants were randomly assigned into three groups of 3 mg·kg^–1^ of BM caffeine ([CAF3], n = 8, age = 21.6 ± 2.5 years, height = 182.6 ± 5.5 cm, weight = 78.5 ± 6.1 kg, and training age = 6.1 ± 1.7 years), 6 mg·kg^–1^ of BM caffeine ([CAF6], n = 8, age = 22.1 ± 1.9 years, height = 183.6 ± 6.2 cm, weight = 81.5 ± 5.3 kg, and training age = 5.9 ± 2.2 years), and placebo ([PL], n = 8, age = 22.8 ± 2.7 years, height = 181.2 ± 5.7 cm, weight = 79.4 ± 5.9 kg, and training age = 5.6 ± 2.9 years). Subjects had to satisfy specific inclusion and exclusion criteria to be eligible for participation in this study: (1) not using any drugs or stimulator supplements (such as power booster) for three months preceding the study; (2) not having any lower body injuries affecting the training and testing sessions; and (3) not being involved in regular plyometric and strength training for three months preceding the study. Additionally, the subjects were considered to be low caffeine consumers (i.e. less than 50 mg of daily caffeine within 2 months prior to their inclusion in the study)^30^, which was confirmed by a valid questionnaire^31^. Prior to any data collection, the participants were fully informed about the potential risks and discomforts associated with the study (Fig. 1). They were required to carefully read and sign the informed consent form, which had been approved by the Ethics Research Committee of the Wuhan Sports University and was in accordance with the Declaration of Helsinki.

### Experimental design

**Figure 2:**
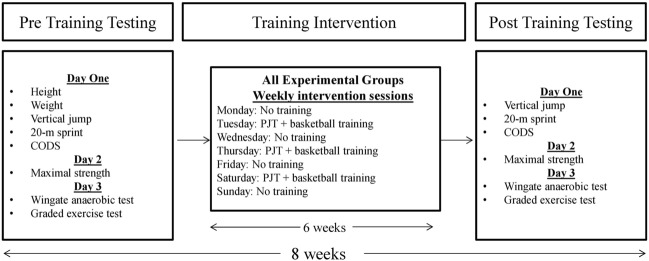
Experimental design.

This study used a randomized, double-blind, placebo-controlled design, which lasted 8 weeks, including 1 week pre-test, 6 weeks PJT, and 1 week post-test. Prior to participation, participants before the baseline measurements, participants made a familiarization visit to the laboratory to become orientated with the testing procedures and training protocols. Before and after the 6-week PJT, participants underwent a series of lab-based tests (i.e. graded exercise test and lower-body Wingate) and field-based teats (i.e. vertical jump, 20-m sprint, Illinois change of direction ability, and maximal strength of the lower-body) to evaluate physiological and sport-specific performance measures (Fig. 2). All subjects engaged in basketball practice on 3 non-consecutive days and completed PJT before their training sessions in the afternoon (from 4:00 to 6:00 P.M.). One hour prior to the training sessions, each subject consumed a capsule containing either 3 or 6 mg·kg^–1^ of BM caffeine or placebo. Participants were asked not to participate in extra training sessions, affecting their study-related outcomes.

### Measurement procedures

The basketball-related physical performance tests were conducted on a wooden basketball court, while physiological parameters were measured in a laboratory that maintained a temperature range of 27–29 °C. Throughout the study, the subjects were instructed to maintain their daily activities. They were advised to refrain from consuming alcohol and caffeine, as well as avoiding intense physical activity within 24 h preceding each testing session. Furthermore, participants were directed to ensure they had a minimum of 9 h of sleep and to wear identical footwear for the pre and post-tests. All testing and training sessions were completed in the same time of the day (4:00 to 6:00 P.M.).

#### Anthropometry

The subjects’ body mass was evaluated using an electrical scale (TANITA, BC-418MA, Tokyo, Japan). Additionally, their height was determined using a stadiometer (SECA, Terre Haute, IN, United States).

#### Vertical jump test

The evaluation of vertical jump (VJ) was carried out following the established procedures. A wall-mounted vertical jump tester (VERTEC Power System, USA) was utilized on the basketball court to measure the VJ. Subjects were instructed to flex their knees to a 90° angle and then perform a jump without using their arms, aiming to reach the maximum height possible. Prior to the VJ performance test, all subjects completed a warm-up consisting of 3 submaximal jumps. This was followed by three maximal jumps for each test, with a 30-s rest period between each attempt. The highest score achieved in each test was selected for subsequent analysis^10^.

#### Sprint and change of direction speed tests

The subjects were given instructions to sprint a distance of 20 m between two electronic timing gates (brower timing systems, Draper, UT, USA, with a nearest of 0.001 s) positioned at the hip level at the start (0 m) and end (20 m) of a linear track on a basketball wooden court. Additionally, the Illinois test was used to evaluate their change of direction speed (CODS). Like the 20-m sprint, the CODS test employed the same timing system and procedures. However, in this test, players ran in a straight line while making multiple direction changes, aiming to complete it as fast as possible. The fastest time achieved out of the three attempts, with a 2-min rest interval between each attempt, was selected for subsequent analysis^12^.

#### Lower-body maximal strength test

The muscular strength of the lower body was evaluated using the leg press device (Body Solid, Model GLPH 1100, USA). The assessment was conducted through the one repetition maximum (1RM) testing, which was carried out following the method previously explained in detail^32^. Briefly, the participants engaged in a warm-up routine for their leg muscles, consisting of three sets of leg presses. The warm-up included one set of 10 repetitions using a light weight, followed by one set of 5 repetitions with a moderate weight, and finally, one set of 3 repetitions with a heavy weight. After completing the warm-up, each participant underwent the 1RM test. During this test, the load was gradually increased in successive trials until the participants were unable to perform a proper lift, complete the full range of motion, or maintain the correct technique. The 1RM value was determined based on approximately five sets of one repetition, with rest periods of 3 to 5 min between each attempt.

#### Graded exercise test

All subjects underwent a graded exercise test on a treadmill (Woodway, Pro Series, Waukesha, WI) until they reached their maximum effort to determine their maximum oxygen consumption (V̇O_2max_)^33^. The initial speed for the graded exercise test was set at 10 km/h with no incline, and it increased by 2 km/h every two minutes until reaching 16 km·h^–1^. After that, the speed increased by 1 km·h^–1^ every minute until 18 km·h^–1^. The incline was then increased by 2% every minute until the subjects reached their V̇O_2max_. To determine V̇O_2max_, open-circuit spirometry was used with a metabolic cart (True One 2400^®^, Sandy, UT) to analyze the breath-by-breath expired gases. Various criteria were employed to determine if the athlete attained V̇O_2max_. These criteria encompassed: (a) the observation of V̇O_2_ leveling off despite an escalation in workload, (b) a respiratory exchange ratio surpassing 1.1, (c) a Bla concentration of 8 mmol·L^–1^ or higher, and (d) a maximum heart rate (Hrmax) equal to or greater than 95% of the age-predicted maximum (220–age)^34–37^.

#### Wingate anaerobic power test

The Wingate test was carried out using a Monark cycle ergometer (Ergomedic 828E, Vansbro, Sweden). Prior to the test, a standardized warm-up, as mentioned in a previous study^34^, was performed. The assessment involved cycling at the utmost exertion for 30 s while carrying a weight equal to 7.5% of the individual’s body weight. The test commenced from a stationary position, and the first pedal stroke was initiated using the dominant leg. Participants were encouraged to rapidly achieve their maximum revolutions per minute (rpm) and maintain this pedaling speed until the assessment was completed. Throughout the test, the investigators motivated the athletes. The power achieved at the 5-s mark and the average power throughout the test were recorded as peak power output (PPO) and average power output (APO), respectively.

### Caffeine supplementation

During the 6-week training period, the CAF3 and CAF6 groups were administered either 3 or 6 mg·kg^–1^ of BM caffeine (100% purity, Bulk Powders, UK), respectively, one hour before each PJT session^21,23^. In contrast, the PL group received an equivalent dose of cellulose, which was also encapsulated. All subjects were instructed to consume the capsules with juice. It is worth noting that the capsules did not contain any information about their composition, ensuring that the investigators and subjects remained unaware of the contents until the end of the study. No symptoms related to side effects of caffeine supplementation were seen throughout the experiment.

### Diet control

The subjects involved in the study were advised to maintain their regular dietary habits without any alterations during the entire study period. Moreover, they had to record their food intake three days before the initial testing and follow the same dietary routine before the post-training test session. The food diaries were analyzed using Nutritionist IV diet analysis software to assess the energy and macronutrient content. The results revealed that the subjects had a similar caloric intake, with 25% protein, 60% carbohydrates, and 15% fat.

### PJT program

The basketball players participated in their regular training sessions on Tuesday, Thursday, and Saturday. These sessions lasted for 70 to 80 min each afternoon, from 4:00 to 6:00 P.M. During these sessions, the players focused on various aspects of the game, including tactical drills, technical exercises, and competitive games. To prepare for the training, the players started with a 15-min warm-up. This warm-up consisted of 5 min of running, 5 min of stretching, and 5 min of sprinting and ballistic movements. Before the basketball training, all the players underwent a PJT program. This program included depth jumps with a 45-cm box height, squat jumps, and knee tuck jumps. The players performed 4 sets of 15 repetitions for each exercise, with 90 and 120 s of rest between sets and exercises^38^. A specialized strength and conditioning coach closely monitored the players throughout the training sessions to ensure that they followed the prescribed training program. This coach played a crucial role in guiding and supporting the athletes during their training.

### Statistical analysis

The mean ± SD was used to present the data. The normality of both pre-and post-values for the dependent variables was assessed using the Shapiro–Wilk Normality test. A repeated-measures ANOVA (3 [group] × 2 [time]) was conducted to identify significant differences between the three groups for each tested variable and Tukey’s post hoc examined interactions or main effects if a significant F-ratio was observed. The effects size (ES), which was determined by Hedges’ g, were categorized as trivial (< 0.20), small (0.20–0.50), medium (0.50–0.80), large (0.80–1.30), or very large (> 1.30). The 95% confidence interval (CI) was also reported^39^. The significance level was set at 0.05. The coefficient of variation (CV) in mean group changes was calculated to assess inter-individual variability. Individual percent changes (∆ %) from pre-training to post-training were calculated for each variable, and these changes’ mean (SD) was determined. The CV (ratio of SD to the mean) of percent changes was then calculated for each variable. Additionally, individual residuals were computed as the square root of the squared difference between individual and mean values for each tested variable. Finally, the impact of interventions on inter-subject variability in variables was evaluated by comparing between-group mean residuals for each variable.

## Results

Throughout the current research, every subject exhibited absolute compliance, leading to a remarkable 100% success rate. Furthermore, no injuries were reported in connection with the training interventions, and subjects did not report experiencing any nervousness or vigor or encountering side effects from consuming CAF. No statistically significant differences (p > 0.05) concerning basketball-related performance and physiological parameters were observed between the groups during the pre-test.

**Figure 3:**
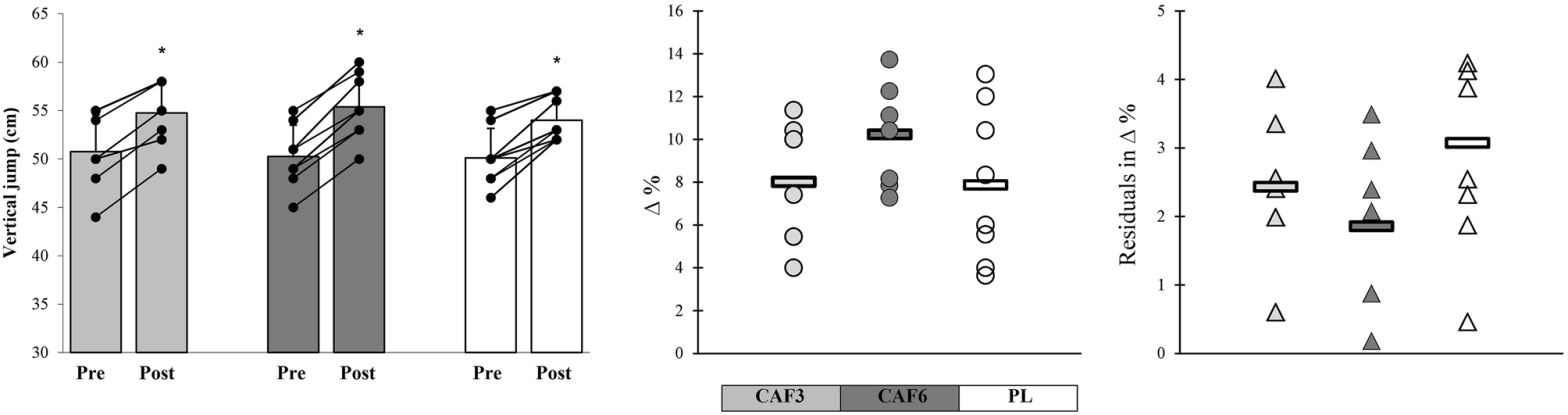
Mean group change, individual changes (∆%), and residuals in ∆% in vertical jump (VJ) in response to CAF3, CAF6, and PL interventions. *Denotes significantly different versus pre-training (p ≤ 0.05). Bar chart present group change (cm) in the measures variables. In the scatter charts, circles and triangles indicate individual percent changes (∆%) from baseline (X-axes), and horizontal bars represent the group percent change (∆%) the training period.

**Figure 4:**
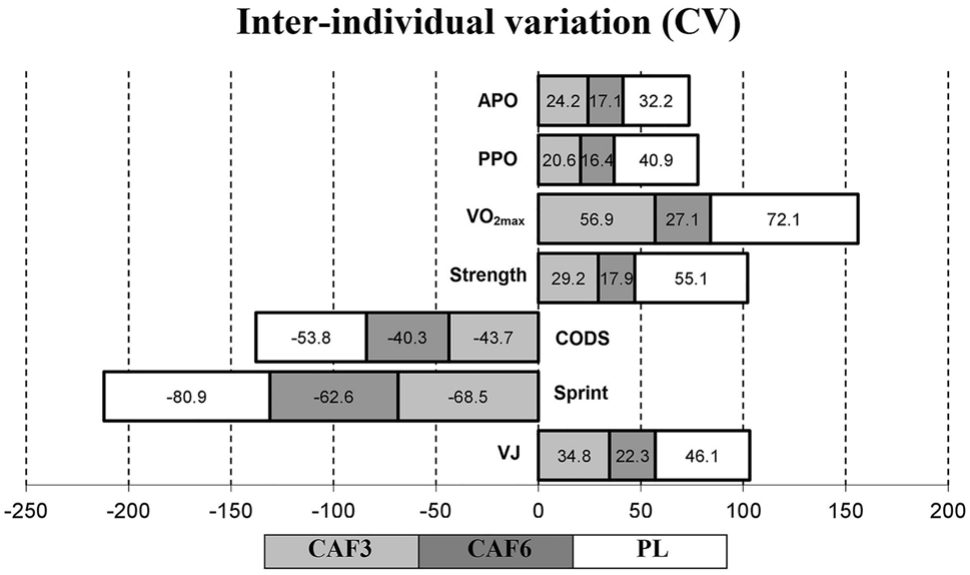
Coefficient of variations (CV) in mean group changes in physiological and performance measures in response to CAF3, CAF6, and PL groups.

Following the 6-week training period, all experimental groups demonstrated significant improvements in the VJ performance (p ≤ 0.05). The CAF6 group exhibited very large meaningful effects (p = 0.002, ES = 1.45, 95% CI 0.35 to 2.55), while the CAF3 (p = 0.001, ES = 1.07, 95% CI 0.02 to 2.11) and PL (p = 0.019, ES = 1.10, 95% CI 0.04 to 2.15) groups showed large meaningful effects on VJ performance. Furthermore, the CAF6 group displayed insignificantly greater adaptations with lower residuals in individual changes and CV in mean group change in VJ compared to the CAF3 and PL groups (Figs. 3 and 4).

**Figure 5:**
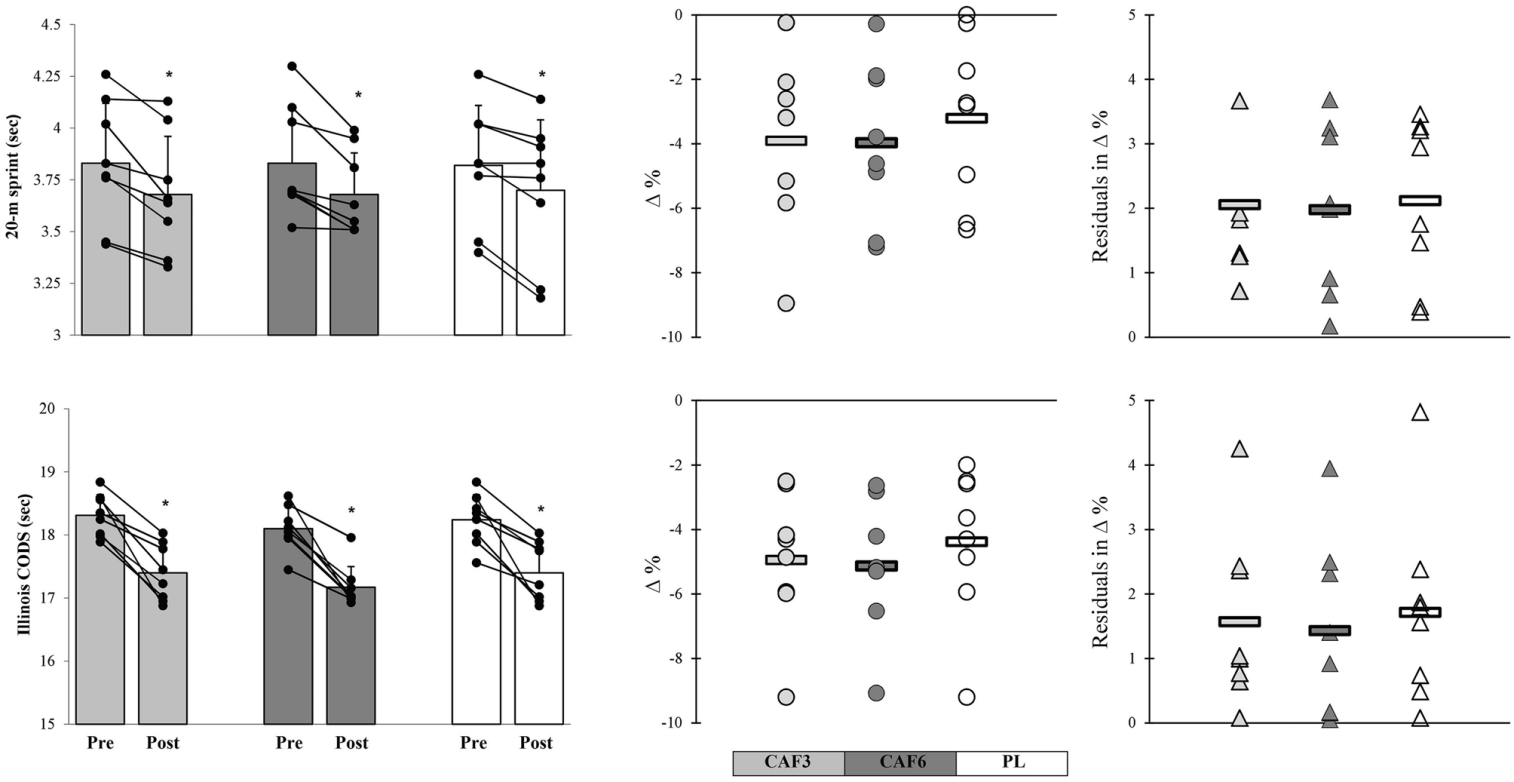
Mean group change, individual changes (∆%), and residuals in ∆% in 20-m sprint, and Illinois CODS in response to CAF3, CAF6, and PL interventions. *Denotes significantly different versus pre-training (p ≤ 0.05). Bar charts present group change (sec) in the measures variables. In the scatter charts, circles and triangles indicate individual percent changes (∆%) from baseline (X-axes), and horizontal bars represent the group percent change (∆%) the training period.

Following the 6-week training period, all experimental groups demonstrated significant improvements in the 20-m sprint (CAF3: p = 0.039, ES = – 0.50, 95% CI – 1.49 to 0.50, Medium, CAF6: p = 0.026, ES = – 0.61, 95% CI – 1.61 to 0.39, Medium, PL: p = 0.047, ES = – 0.36, 95% CI – 1.35 to 0.63, Small) and Illinois CODS (CAF3: p = 0.025, ES = – 1.22, 95% CI – 2.28 to – 0.15, Large, CAF6: p = 0.017, ES = – 1.26, 95% CI – 2.33 to – 0.18, Large, PL: p = 0.05, ES = – 1.09, 95% CI – 2.14 to − 0.04, Large). Upon comparing percent changes and individual residuals in changes, the CAF6 group indicated insignificantly greater adaptive responses with lower residuals in individual changes and CVs in mean group changes compared to the CAF3 and PL groups (Figs. 4 and 5).

**Figure 6:**
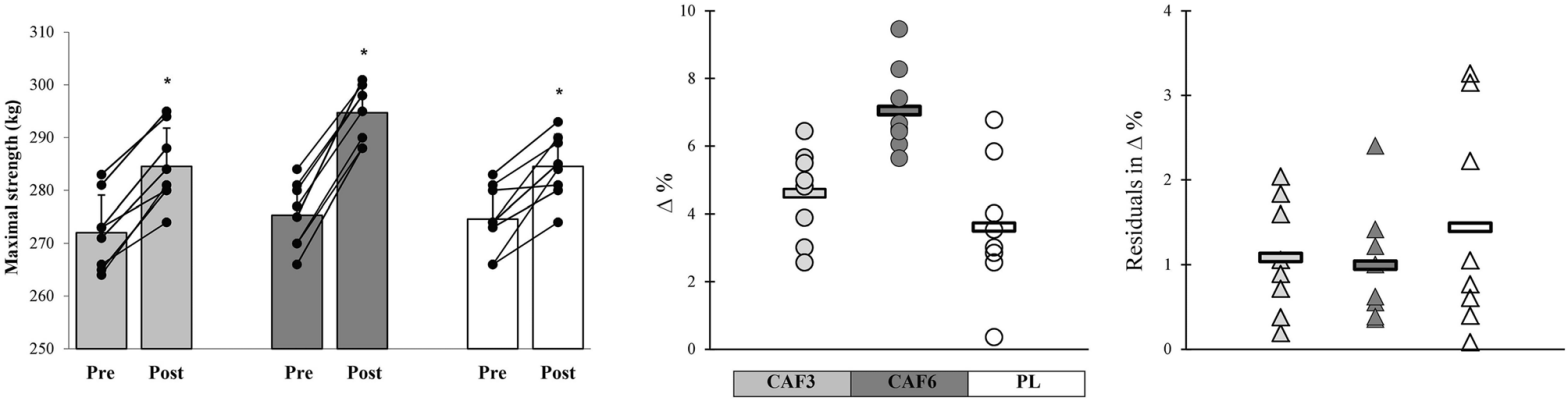
Mean group change, individual changes (∆%), and residuals in ∆% in maximal strength in response to CAF3, CAF6, and PL interventions. *Denotes significantly different versus pre-training (p ≤ 0.05). Bar chart present group change (kg) in the measures variables. In the scatter charts, circles and triangles indicate individual percent changes (∆%) from baseline (X-axes), and horizontal bars represent the group percent change (∆%) the training period.

All experimental groups significantly improved maximal strength over time (p ≤ 0.05). The CAF3 (p = 0.001, ES = 1.68, 95% CI 0.51 to 2.77) and CAF6 (p = 0.001, ES = 2.29, 95% CI 1.03 to 3.55) groups exhibited very large, meaningful effects, while the PL (p = 0.049, ES = 1.17, 95% CI 0.11 to 2.23) group showed large, meaningful effects on strength performance after training. Furthermore, the CAF6 group displayed insignificantly greater adaptive responses with lower residuals in changes and CV in mean group change compared to the CAF3 and PL groups (Figs. 4 and 6).

**Figure 7:**
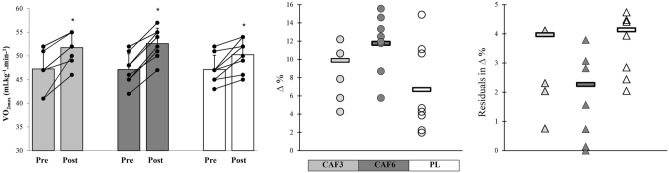
Mean group change, individual changes (∆%), and residuals in ∆% in V̇O_2max_ in response to CAF3, CAF6, and PL interventions. *Denotes significantly different versus pre-training (p ≤ 0.05). Bar chart present group change (ml·kg·min^–1^) in the measures variables. In the scatter charts, circles and triangles indicate individual percent changes (∆%) from baseline (X-axes), and horizontal bars represent the group percent change (∆%) the training period.

V̇O_2max_ was significantly enhanced in response to all training interventions from pre- to post-training (p ≤ 0.05). The CAF6 group exhibited very large meaningful effects (p = 0.001, ES = 1.59, 95% CI 0.46 to 2.71), while the CAF3 (p = 0.001, ES = 1.09, 95% CI 0.04 to 2.14) and PL (p = 0.036, ES = 0.92, 95% CI – 0.11 to 1.95) groups showed large meaningful effects in enhancing V̇O_2max_ after training. Furthermore, the CAF6 group displayed insignificantly greater adaptive responses with lower residuals in individual changes and CV in mean group change compared to the CAF3 and PL groups (Figs. 4 and 7).

**Figure 8:**
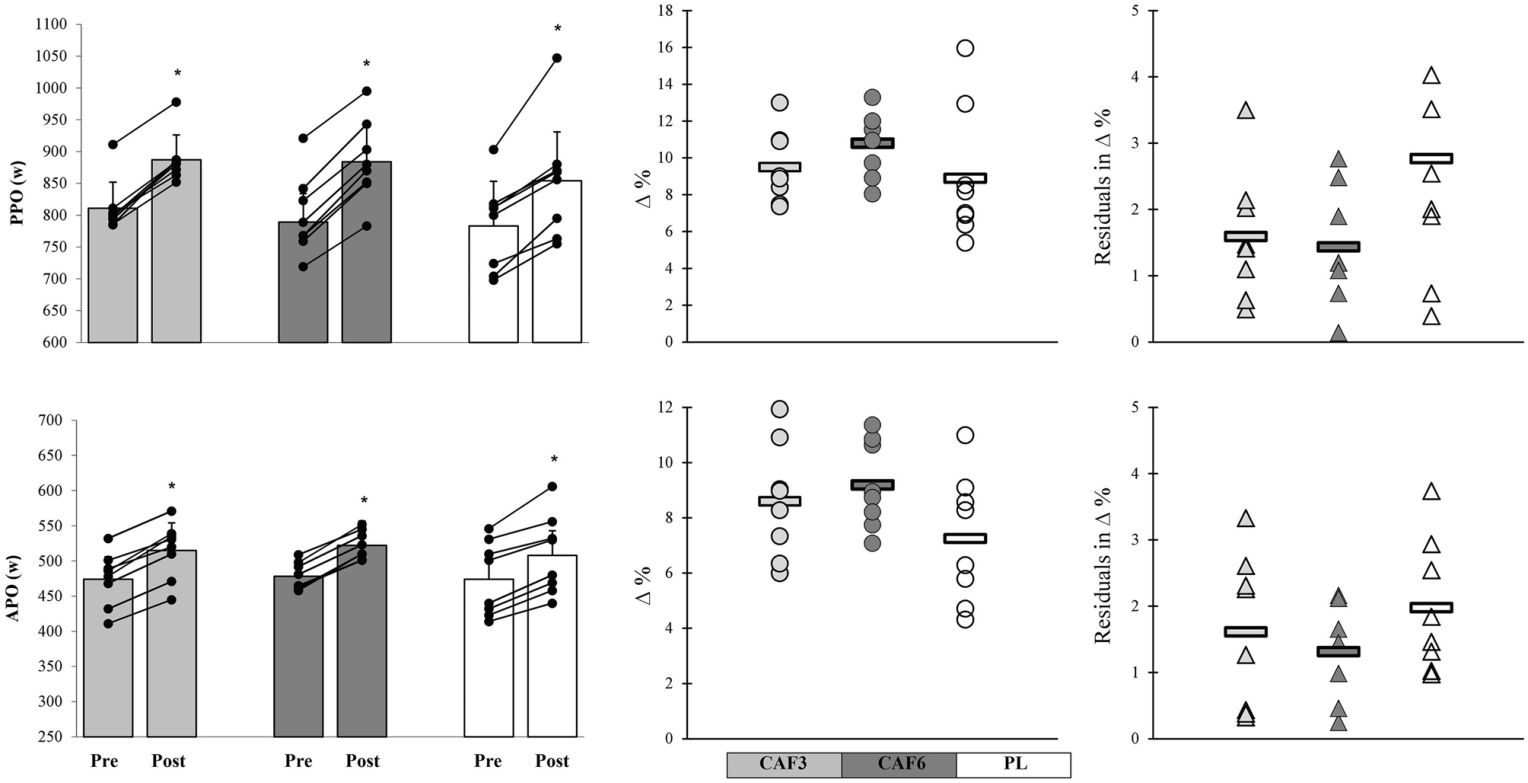
Mean group change, individual changes (∆%), and residuals in ∆% in PPO, and APO in response to CAF3, CAF6, and PL interventions. *Denotes significantly different versus pre-training (p ≤ 0.05). Bar chart present group change (W) in the measures variables. In the scatter charts, circles and triangles indicate individual percent changes (∆%) from baseline (X-axes), and horizontal bars represent the group percent change (∆%) the training period.

All interventions significantly enhanced PPO and APO from pre- to post-training (p ≤ 0.05). The CAF3 and CAF6 groups exhibited very large meaningful effects on the PPO (CAF3, p = 0.001, ES = 1.82, 95% CI 0.65 to 2.98; CAF6, p = 0.001, ES = 1.85, 95% CI 0.68 to 3.02), and APO (CAF3, p = 0.001, ES = 1.31, 95% CI 0.20 to 2.42; CAF6, p = 0.001, ES = 1.39, 95% CI 0.30 to 2.38), while the PL group showed large, meaningful effects on the PPO (p = 0.036, ES = 0.82, 95% CI – 0.20 to 1.84), and APO (p = 0.006, ES = 1.07, 95% CI 0.02 to 2.12). Furthermore, the CAF6 group displayed insignificantly greater adaptive responses with lower residuals in individual changes and CV in mean group change compared to the CAF3 and PL groups (Figs. 4 and 8).

## Discussion

This study was designed to determine the optimal dose of CAF (3 or 6 mg·kg^–1^ of BM) ingested prior to PJT on physiological and performance adaptations of collegiate basketball athletes over a 6-week training period. We also investigated individual adaptive responses to the mentioned interventions. Our results indicated that consumption of CAF prior to PJT is a viable strategy for inducing greater adaptive changes than the PJT alone. In addition, 6 mg·kg^–1^ of CAF supplementation produced more adaptive advantages in both the performance and physiological parameters with greater uniformity of adaptations across participants.

In this study, all subjects increased their VJ ability following the 6 weeks of PJT, ranging between large to very large ES. The improvements in VJ of basketball athletes following the PJT have been reported in previous studies with large ES^10,12,40,41^, which aligns with our findings. Various adaptive mechanisms contribute to the improvements in jumping performance achieved with PJT. These mechanisms include enhanced recruitment of motor units, improved coordination between different muscles, increased neural drive to the agonist muscles, and optimized utilization of the SSC^42^.

When examining the effects of caffeine consumption before PJT, it was found that a higher dosage of caffeine intake resulted in more adaptive changes in the VJ. The group that consumed 6 mg·kg^–1^ of BM caffeine experienced very large training benefits, while the CAF3 and PL groups demonstrated large training effects. Moreover, the CAF6 group exhibited a higher percentage of change with lower residuals in individual changes and CV in mean group change than the other groups. It seems ingesting 6 mg·kg^–1^ of BM caffeine could induce greater adaptive changes in neuromuscular adaptations, resulting in greater changes in VJ performance^23^. Additional suggested mechanisms induced by caffeine could be highlighted by the release of ß-endorphins^23^ or increases in intracellular calcium mobilization^20^. Nevertheless, current understanding suggests that the majority of caffeine’s performance-enhancing properties are attributed to its impact on the central nervous system via adenosine receptor antagonism^21^. Adenosine and caffeine exert contrasting effects on cellular activities^20^. Consequently, caffeine consumption enhances the influence of adenosine on neurotransmission, perceived exertion, and arousal during the training period^19,23^, inducing more work in the muscle fibers by PJT, leading to improved VJ performance.

PJT also significantly enhanced sprint performance and CODS ability of basketball players with small to large training effects, which are in accordance with previous studies in basketball^12,14,40,41^. Increases in these qualities following PJT may be attributed to “enhanced neuromuscular activation of the muscles including an increase in the number of firing frequencies in activated motor units, changes in the recruitment pattern of motor units, particularly in fast-twitch muscle fibers”^12,15,43–45^. Furthermore, lower body PJT induces neuromechanical adaptations such as improved neural drive to agonist muscles and optimization of muscle–tendon stiffness, which can enhance the effectiveness of the SSC and eccentric workload that are vital for enhancing sprint and CODS^43,44^.

When comparing the impact of consuming different dosages of CAF prior to PJT, it was observed that both the CAF6 and CAF3 groups resulted in similar improvements in the 20-m sprint and Illinois CODS. It appears that a dosage of 3 or 6 mg·kg^–1^ of BM caffeine is the optimal amount to induce adaptations in sprint and CODS. Higher doses do not seem to have any additional effects. The similarity in the activation of motor units resulting from ingesting of 3 and 6 mg of caffeine prior to PJT^23^ may explain the similar improvements observed in athletes’ performance in bio-motor abilities, including sprinting and CODS^15^. However, additional studies are needed to elucidate the specific adaptations required to enhance sprint and CODS performance through the consumption of different doses of caffeine.

Regarding the strength performance, all the training groups displayed significant improvements compared to baseline values. The CAF6 and CAF3 groups indicated very large ES, while the PL group showed large ES after the 6-week PJT. The findings of this study support the previous meta-analysis and experimental studies that examined the benefits of PJT on maximal strength in individuals and found positive transfer of PJT to enhance maximal strength^40,41,46^. It is suggested that the improvements in strength observed with PJT may be attributed to “neural adaptations, including enhanced motor-unit firing frequency, synchronization, excitability, and efferent motor drive”^46^. The relative force generated by each recruited motor unit can be optimized through these adaptive mechanisms^41^. It is important to mention that muscle hypertrophy may also contribute to the enhancement of muscle strength following PJT^40^.

The CAF6 group exhibited greater g”Ins ’n percent change and lower residuals in changes and CV compared to the other groups. Based on the findings, it can be concluded that the consumption of higher dosages of caffeine before engaging in PJT has an antagonistic impact on adenosine receptors in both the central and peripheral nervous systems^23^. CAF enhances central drive and reduces the sensation of pain and fatigue during exercise^19^. Additionally, caffeine stimulates the release of serotonin in the cerebral cortex, exerting its influence on the central nervous system^20^. This, in turn, enhances the performance of the sympathetic system while reducing the activity of inhibitory neurons^20^. As a result, PJT is performed by higher arousal, leading to greater activation of muscle fibers, resulting in greater strength gains with more homogeneity in adaptations.

In this study, a novel approach was taken to investigate the effects of PJT on the cardiorespiratory fitness (V̇O_2max_) of basketball athletes. The findings revealed that PJT is an optimal method for enhancing V̇O_2max_, as it induced significant improvements. Interestingly, ingesting low doses of caffeine did not lead to greater gains than a placebo, while high doses of caffeine resulted in even greater adaptive changes than other groups. Typically, cardiorespiratory fitness improvements through physical training is mediated by adaptations in both central and peripheral systems^47–49^, including improved oxygen delivery and utilization by the active muscles during exercise^50^. However, the ingestion of caffeine prior to PJT showed even more promising results, suggesting that a 6 mg·kg^–1^ of BM caffeine could be advantageous for basketball athletes in promoting their cardiorespiratory fitness over a 6-week duration.

The enhancement of V̇O_2max_ can be attributed to several mechanisms induced by caffeine ingestion. One such mechanism involves the breakdown of caffeine, resulting in the production of theobromine^23^. Acting as a vasodilator, theobromine increases the flow of oxygen and nutrients to the brain and muscles^23^. Another component of caffeine, theophylline, enhances the efficiency of the heart^21^. Additionally, paraxanthine increases the presence of glycerin and fatty acids in the bloodstream, which serve as an energy source for the muscles^20^. Furthermore, caffeine aids in releasing calcium, which plays a role in converting and secreting glycogen phosphorylase B-enzyme^21^. As a result, these pathways enable individuals to engage in aerobic exercise with a lower perceived exertion and fatigue^23^, ultimately leading to more extended time to exhaustion and greater improvements in cardiorespiratory fitness, specifically V̇O_2max_.

Throughout the 6-week training duration, incorporating PJT yielded substantial increments in PPO and APO among all athletes. The administration of both caffeine doses exhibited very large significant improvements in PPO and APO, whereas the PL group displayed large significant training effects. PJT represents a favorable training technique for enhancing the power performance of basketball players by promoting neural and morphological adaptations through the augmentation of anabolic hormonal concentrations^41,43^. PJT stimulated increased motor unit recruitment and forceful muscular contraction, ultimately leading to enhancements in power output^10^.

Caffeine consumption before PJT resulted in greater gains in PPO and APO when 3 or 6 mg·kg^–1^ of BM was ingested. However, the CAF6 group showed slightly greater gains with lower residual in individual changes and CV in mean group change compared to the other supplemented group. This suggests that caffeine is a suitable strategy to promote homogeneity in power adaptations among basketball players. Caffeine stimulates the sarcoplasmic reticulum to release more calcium, thereby increasing the recruitment of motor units^30^. Consequently, this leads to more forceful muscular contractions, which can explain some of the ergogenic effects observed in power-based performance. The enhancements in PPO and APO following caffeine ingestion can be attributed to their impact on the A1, A2A, and A2B adenosine receptors^21^. Adenosine inhibits efferent nerve activity during exercise, while caffeine promotes the synthesis of neurotransmitters that stimulate the brain, resulting in increased alertness and improved mood^23^. This enhanced vigor and tension are crucial for power performance^20^. Caffeine ingestion before PJT stimulates adenosine antagonism and facilitates neuromuscular recruitment, reducing the time required to reach peak power levels^19^. This likely contributes to the higher power levels observed in our study. Consequently, consuming caffeine before PJT induces adaptations in power performance and promotes uniformity in power performance gains, which is essential for coaches aiming to improve their players’ performance consistently during the training period.

One limitation of this study was its inability to account for genetic variations among participants, which are recognized to impact trainability and adaptive response, especially concerning V̇O_2max_. Moreover, the study lacked close monitoring of factors such as sleep quality, dietary habits, and psychological aspects like motivation. While participants were advised to maintain consistency, strict control over these variables remained unattainable. Lastly, the study’s participant group consisted solely of males, which restricts the generalizability of the findings to female swimmers.

## Conclusions

The results of the current study indicate that PJT is a viable approach for enhancing the performance and physiological parameters of male basketball players. Furthermore, the ingesting 6 mg·kg^–1^ caffeine can promote uniformity of adaptations in cardiorespiratory fitness, anaerobic performance, and sport-related performance measures across basketball players. Based on these findings, it is advisable for basketball coaches, trainers, and athletes to consider incorporating CAF (at a dosage of 6 mg·kg^–1^ of BM) as an ergogenic aid to induce greater adaptive responses and uniform adaptations in the variables related to basketball performance.

## Practical applications

This study compared the effects of ingesting different caffeine dosages (3 or 6 mg·kg^–1^ of BM) before plyometric jump training on sport-specific bio-motor abilities, aerobic fitness, and anaerobic power in male basketball players. Our results indicate that both interventions adequately stimulate mechanisms involved in the adaptation in variables representing the qualities mentioned above. However, based on the lower residuals in the individual adaptive changes in measured parameters in response to CAF6 intervention and its insignificantly greater effect (ES) on jumping ability, maximal strength, and V̇O_2max_, coaches, and athletes can consider this intervention for inducing more uniform homeostatic stress across team members and to get more desired outcomes in the parameters mentioned above.

## Data Availability

The datasets employed and/or analyzed in the current study can be provided upon a reasonable request from the corresponding author.

## References

[1] Simenz CJ, Dugan CA, Ebben WP (2005). Strength and conditioning practices of National Basketball Association strength and conditioning coaches. J. Strength Cond. Res..

[2] Stojanović E, Stojiljković N, Scanlan AT, Dalbo VJ, Berkelmans DM, Milanović Z (2018). The activity demands and physiological responses encountered during basketball match-play: A systematic review. Sports Med..

[3] Scanlan AT, Dascombe BJ, Kidcaff AP, Peucker JL, Dalbo VJ (2015). Gender-specific activity demands experienced during semiprofessional basketball game play. Int. J. Sports Physiol. Perform..

[4] Chaouachi A (2009). Lower limb maximal dynamic strength and agility determinants in elite basketball players. J. Strength Cond. Res..

[5] Sperlich PF, Behringer M, Mester J (2016). The effects of resistance training interventions on vertical jump performance in basketball players: A meta-analysis. J. Sports Med. Phys. Fit..

[6] Ziv G, Lidor R (2010). Vertical jump in female and male basketball players—A review of observational and experimental studies. J. Sci. Med. Sport.

[7] Gleddie N, Marshall D (1996). Plyometric training for basketball. Strength Cond. J..

[8] Markovic G, Mikulic P (2010). Neuro-musculoskeletal and performance adaptations to lower-extremity plyometric training. Sports Med..

[9] Bobbert MF (1990). Drop jumping as a training method for jumping ability. Sports Med..

[10] Matavulj D, Kukolj M, Ugarkovic D, Tihanyi J, Jaric S (2001). Effects of plyometric training on jumping performance in junior basketball players. J. Sports Med. Phys. Fit..

[11] Mancha-Triguero D, García-Rubio J, Calleja-González J, Ibáñez SJ (2019). Physical fitness in basketball players: A systematic review. J. Sports Med. Phys. Fit..

[12] Arazi H, Coetzee B, Asadi A (2012). Comparative effect of land- and aquatic-based plyometric training on jumping ability and agility of young basketball players. S. Afr. J. Res. Sport Phys. Educ. Recreat..

[13] Wilkerson GB, Colston MA, Shortt NI, Neal KL, Hoewischer PE, Pixley JJ (2004). Neuromuscular changes in female collegiate athletes resulting from a plyometric jump-training program. J. Athl. Train..

[14] Arazi H, Asadi A (2011). The effect of aquatic and land plyometric training on strength, sprint, and balance in young basketball players. J. Hum. Sport Exerc..

[15] Asadi A, Arazi H, Ramirez-Campillo R, Moran J, Izquierdo M (2017). Influence of maturation stage on agility performance gains after plyometric training: A systematic review and meta-analysis. J. Strength Cond. Res..

[16] Delleli S, Ouergui I, Messaoudi H, Ballmann CG, Ardigò LP, Chtourou H (2023). Effects of caffeine consumption combined with listening to music during warm-up on taekwondo physical performance, perceived exertion and psychological aspects. PLoS One.

[17] Ouergui I, Delleli S, Bridge CA, Messaoudi H, Chtourou H, Ballmann CG, Ardigò LP, Franchini E (2023). Acute effects of caffeine supplementation on taekwondo performance: The influence of competition level and sex. Sci. Rep..

[18] Ouergui I, Mahdi N, Delleli S, Messaoudi H, Chtourou H, Sahnoun Z, Bouassida A, Bouhlel E, Nobari H, Ardigò LP, Franchini E (2022). Acute effects of low dose of caffeine ingestion combined with conditioning activity on psychological and physical performances of male and female taekwondo athletes. Nutrients.

[19] Barzegar H, Arazi H, Mohebbi H, Sheykhlouvand M, Forbes SC (2022). Caffeine co-ingested with carbohydrate on performance recovery in national-level paddlers: A randomized, double-blind, crossover, placebo-controlled trial. J. Sports Med. Phys. Fit..

[20] Nemati J (2023). Effects of different doses of caffeine supplementation on collegiate male volleyball players’ specific performance and skills: A randomized, double-blind, placebo-controlled, crossover study. Nutrients.

[21] Castillo D, Domínguez R, Rodríguez-Fernández A, Raya-González J (2019). Effects of caffeine supplementation on power performance in a flywheel device: A randomized, double-blind cross-over study. Nutrients.

[22] Forbes SC, Sheykhlouvand M (2016). A review of the physiological demands and nutritional strategies for canoe polo athletes. J. Nutr. Sci. Res..

[23] Grgic J, Trexler ET, Lazinica B, Pedisic Z (2018). Effects of caffeine intake on muscle strength and power: A systematic review and meta-analysis. J. Int. Soc. Sports Nutr..

[24] Matsumura T, Tomoo K, Sugimoto T, Tsukamoto H, Shinohara Y, Otsuka M, Hashimoto T (2023). Acute effect of caffeine supplementation on 100-m sprint running performance: A field test. Med. Sci. Sports Exerc..

[25] Horiuchi M, Nagahara R (2024). Acute effects of caffeine supplementation on kinematics and kinetics of sprinting. Scand. J. Med. Sci. Sports.

[26] Duvnjak-Zaknich DM, Dawson BT, Wallman KE, Henry G (2011). Effect of caffeine on reactive agility time when fresh and fatigued. Med. Sci. Sports Exerc..

[27] Doherty M, Smith PM (2004). Effects of caffeine ingestion on exercise testing: A meta-analysis. Int. J. Sport Nutr. Exerc. Metab..

[28] Gharaat MA, Sheykhlouvand M, Eidi LA (2020). Performance and recovery: Effects of caffeine on a 2000-m rowing ergometer. Sport Sci. Health.

[29] Faul F, Erdfelder E, Lang AG, Buchner A (2007). G*Power 3: A flexible statistical power analysis program for the social, behavioral, and biomedical sciences. Behav. Res. Methods.

[30] Filip A (2020). Inconsistency in the ergogenic effect of caffeine in athletes who regularly consume caffeine: Is it due to the disparity in the criteria that defines habitual caffeine intake?. Nutrients.

[31] Bühler E (2013). Development of a tool to assess the caffeine intake among teenagers and young adults. Ernahrungs Umschau.

[32] Kraemer WJ, Fry AC (1995). ACSM’s Guidelines for Exercise Testing and Prescription.

[33] Alejo LB (2022). Comparative analysis of endurance, strength and body composition indicators in professional, under-23 and junior cyclists. Front. Physiol..

[34] Sheykhlouvand M, Gharaat M (2024). Optimal homeostatic stress to maximize the homogeneity of adaptations to interval interventions in soccer players. Front. Physiol..

[35] Fereshtian S, Sheykhlouvand M, Forbes S, Agha-Alinejad H, Gharaat M (2017). Physiological and performance responses to high-intensity interval training in female inline speed skaters. Apunts Med. l’Esport.

[36] Sheykhlouvand M, Forbes SC (2017). Aerobic capacities, anaerobic power, and anthropometric characteristics of elite female canoe polo players based on playing position. Sport Sci. Health.

[37] Sheykhlouvand M, Gharaat M, Bishop P, Khalili E, Karami E, Fereshtian S (2015). Anthropometric, physiological, and performance characteristics of elite canoe polo players. Psychol. Neurosci..

[38] Chu DA (1998). Jumping into Plyometric.

[39] Seitz LB, Reyes A, Tran TT, Saez de Villarreal ES, Haff GG (2014). Increases in lower-body strength transfer positively to sprint performance: A systematic review with meta-analysis. Sports Med..

[40] Ramirez-Campillo R, García-Hermoso A, Moran J, Chaabene H, Negra Y, Scanlan AT (2022). The effects of plyometric jump training on physical fitness attributes in basketball players: A meta-analysis. J. Sport Health Sci..

[41] Asadi A, Ramirez-Campillo R, Meylan C, Nakamura FY, Cañas-Jamett R, Izquierdo M (2017). Effects of volume-based overload plyometric training on maximal-intensity exercise adaptations in young basketball players. J. Sports Med. Phys. Fit..

[42] de Villarreal ES, Kellis E, Kraemer WJ, Izquierdo M (2009). Determining variables of plyometric training for improving vertical jump height performance: A meta-analysis. J. Strength Cond. Res..

[43] Asadi A (2013). Effects of in-season short-term plyometric training on jumping and agility performance of basketball players. Sport Sci. Health.

[44] Asadi A (2013). Effects of in-season plyometric training on sprint and balance performance in basketball players. Sport Sci..

[45] Fachina R (2017). Combined plyometric and strength training improves repeated sprint ability and agility in young male basketball players. Gazz. Med. Ital. Arch. Sci. Med..

[46] de Villarreal ES, Requena B, Newton RU (2010). Does plyometric training improve strength performance? A meta-analysis. J. Sci. Med. Sport.

[47] Sheykhlouvand M, Khalili E, Gharaat M, Arazi H, Khalafi M, Tarverdizadeh B (2018). Practical model of low-volume paddling-based sprint interval training improves aerobic and anaerobic performances in professional female canoe polo athletes. J. Strength Cond. Res..

[48] Rasouli Mojez M, Gaeini AA, Choobineh S, Sheykhlouvand M (2021). Hippocampal oxidative stress induced by radiofrequency electromagnetic radiation and the neuroprotective effects of aerobic exercise in rats: A randomized control trial. J. Phys. Act. Health.

[49] Sayevand Z, Nazem F, Nazari A, Sheykhlouvand M, Forbes SC (2022). Cardioprotective effects of exercise and curcumin supplementation against myocardial ischemia–reperfusion injury. Sport Sci. Health.

[50] Sheykhlovinad M, Khalili E, Gharaat M, Arazi H, Khalafi M, Tarverdizadeh B (2018). Practical model of low-volume paddling-based sprint interval training improves aerobic and anaerobic performances in professional female Canoe Polo athletes. J. Strength Cond. Res..

